# Twenty-first century: the beginning of the replacement parts era?

**DOI:** 10.1590/S1516-31802012000200001

**Published:** 2012-04-03

**Authors:** Alessandro Wasum Mariani, Paulo Manuel Pêgo-Fernandes

**Affiliations:** I MD. Thoracic Surgeon, Instituto Dante Pazzanese de Cardiologia, and Postgraduate Student in the Discipline of Thoracic and Cardiovascular Surgery, Faculdade de Medicina da Universidade de São Paulo (FMUSP).; II MD, PhD. Associate Professor, Discipline of Thoracic Surgery, Instituto do Coração (InCOR), Hospital das Clínicas (HC), Faculdade de Medicina da Universidade de São Paulo (FMUSP), São Paulo, Brazil.

For many years, science fiction explored the idea of treating diseases by means of using artificial devices. References to replacement of human organs by implantable mechanical devices can be found in many books and films. The advances within medicine and bioengineering that have been achieved between the end of the twentieth century and the present date seem to be turning this dream into reality.

There are now six indexed scientific journals specifically focusing on this topic (Artificial Organs; Journal of Artificial Organs; Artificial Organs Today; Biomaterials, Artificial Cells, and Artificial Organs; Biomaterials, Medical Devices and Artificial Organs; and Trends in Biomaterials and Artificial Organs), and there is absolutely no doubt that this exemplifies the importance of this subject. Another indication of the seriousness of such research is that a search in the PubMed database using the key word “Artificial Organs” produces 43,411 retrieved results.

The interest in this field has grown as materials with greater biocompatibility have become available and as device miniaturization has progressively accelerated.^1^ Private initiative, separately or in association with university departments and research institutes, has funded a good proportion of this research, which has already resulted in patents and commercially marketed devices. The need for specialized professionals, whether as physicians, engineers, biologists or bioengineers, is growing and thus opening up new avenues for these professions.

Among the organs that might be replaced, research is advancing particularly in relation to development of an artificial heart, kidney replacement therapy using “portable” machines, replacement limb orthoses and devices performing the endocrine function of the pancreas.

Dialysis is the most widely used form of replacement of internal organ function with an artificial device. However, the need for dialysis sessions in which the patient remains literally a prisoner of the machine has a major impact on the quality of life of patients with kidney failure. Miniaturization of dialysis systems has now made it possible to construct and, on an experimental basis, to use portable dialysis devices that can be attached to the patient’s body, thus allowing some activities to be performed during the dialysis period. In 2008, Gura et al. published a paper on a non-implantable dialysis device that was adapted to be inserted in a vest, thereby providing the patient with full mobility.^2^

Development of an artificial heart has now been studied for approximately 50 years. Today, this research has given rise to several long-term devices known as VADs (ventricular assist devices), which can be implanted. These devices enable life support through increased cardiac function for prolonged periods. Among these, the ones that have been most studied are: DeBakey VAD (MicroMed, Houston, United States), HeartMate II (Thoratec, Pleasanton, United States), DuraHeart (Terumo, Ann Arbor, United States), Incor (Berlin Heart, Germany) and HeartWare HVAD (HeartWare, Framingham, United States).

Although complete artificial hearts now exist, the problems inherent to such devices (need for anticoagulation and battery life, among others) have so far meant that they are not used as the definitive treatment for most patients. Their use is often as a bridge to transplantation.^3^ Nevertheless, since research is heading towards solutions for these problems, many investigators believe that such devices will be increasingly used in a definitive manner, within a few years. Another important point is that studies have already demonstrated that these devices, even with their limitations, have the capacity to increase not only the survival but also the quality of life of patients with advanced heart diseases.^4^

Refinement of the bioengineering will also make it possible for organs with endocrine functions to be replaced. The best examples are systems capable of replacing the endocrine function of the pancreas. Devices known as “insulin bombs”, which have been developed and are already in clinical use, consist of microprocessor systems that measure blood glucose levels and automatically respond with insulin infusions. No implantable models are currently commercially available, but the progressive reduction in volume and increase in precision of these devices indicate that, in the near future, an “implantable artificial endocrine pancreas” may become available for treating diabetes mellitus.^5^

Although ventilatory support and oxygenation machines (extracorporeal circulation) are well-known replacements for lung function, no prototypes for lungs that could be implantable yet exist. Nonetheless, the emergence of smaller-sized devices such as the New Lung,^6^ along with advances in tissue bioengineering, can be expected to motivate researchers in this field.

Medicine and engineering are rapidly and consistently advancing together within the field of development of artificial organs. Recent papers like those described here signal that we will soon have new therapeutic alternatives available. However, the real impact of these devices on longevity and quality of life among the population can be expected to remain unknown for many years. The high estimated cost of using these products and what this will represent for the already far too high cost of modern medicine is the most worrying factor.
